# Research Progress on Flowering Period of Hemp

**DOI:** 10.3390/plants15050682

**Published:** 2026-02-25

**Authors:** Lie Yang, Chao Fan, Jiaxi Li, Hongmei Yuan, Lili Cheng, Dandan Liu, Wenyuan He, Qinghua Kang, Xixia Song, Dandan Yao, Weidong Jiang, Wenjie Zhang, Lili Tang

**Affiliations:** 1 Institute of Industrial Crops of Heilongjiang Academy of Agricultural Sciences, Harbin 150001, China; yanglie1999@126.com (L.Y.); a1597047282@outlook.com (X.S.);; 2 Institute of Tillage and Cultivation of Heilongjiang Academy of Agricultural Sciences, Harbin 150001, China; 3 Key Laboratory of Biology and Genetic Improvement of Horticultural Crops (Northeast Region), College of Horticulture and Landscape Architecture, Northeast Agricultural University, Harbin 150030, China; 4 College of Modern Agriculture and Ecological Environment, Heilongjiang University, Harbin 150080, China; 2232234@s.hlju.edu.cn

**Keywords:** *Cannabis sativa* L., flowering regulation, molecular mechanism, research progress

## Abstract

Flowering regulation in hemp is critical for determining fiber yield, seed production, and the accumulation of medicinal components. This paper, based on bibliometric analysis, highlights the current gap in basic research on cannabis floral organs. The latest advancements in flowering regulation are then systematically reviewed. The morphological and physiological foundations of flowering are examined, including the flowering phenotype, timing, and flower differentiation. Furthermore, the direct regulatory mechanisms of key environmental and cultivation factors—such as photoperiod (type, light quality, duration) and plant nutrition (fertilization, hormones)—on flowering are discussed. Potential pathways through which biotic and abiotic stresses indirectly affect flowering by disrupting metabolic processes are also explored. In addition, the genetic basis of flowering regulation, including key gene loci such as *Autoflower1*, *Early1*, and *CsPRR37*, as well as molecular networks like the FT-mediated photoperiod pathway and the miR156-SPL age pathway, is examined in detail. Finally, the industrial significance of flowering regulation is summarized, and future research directions are proposed to provide a theoretical foundation for the precise breeding and cultivation management of high-quality hemp varieties.

## 1. Introduction

*Cannabis sativa* L. is an annual herb from the genus *Cannabis*, typically diploid in nature (2*n* = 20) [1], with a long history of cultivation [2]. It is extensively utilized across various industries, including textiles, medicine, chemicals, construction, food, and healthcare [3]. As the scope of cannabis applications continues to expand, key industrial objectives have emerged: improving yield stability, optimizing medicinal component accumulation, and enhancing regional adaptability. In this context, the flowering period plays a pivotal role in regulating the plant’s growth cycle, photosynthate distribution, and the formation of final economic traits. Therefore, precise regulation of flowering is essential for achieving these industrial goals [4].

**Figure 1:**
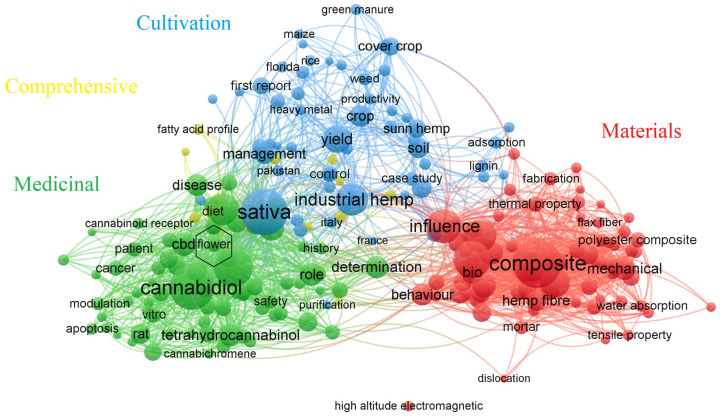
Keyword co-occurrence network of cannabis research based on the Web of Science database. Note: The node size represents keyword frequency, and connections indicate co-occurrence intensity. The black marker hexagon is marked as the theme ‘flower’. Different colors of nodes correspond to four thematic clusters: Medicinal (green), Materials (red), Cultivation (blue), and Comprehensive (yellow).

The flowering period of hemp is a complex trait influenced by genetic, environmental, and cultivation factors. Genetically, flowering time varies among different genotypes [5]. Environmental and cultivation factors such as photoperiod [6], temperature [7], nutrient supply [8], and exogenous hormones significantly impact the initiation and progression of flowering [9]. Although studies in model plants, such as *Arabidopsis* and soybeans, offer valuable insights [10,11], the current research on cannabis flowering primarily focuses on isolated regulatory factors (e.g., photoperiod and hormones), often lacking systematic integration and being fragmented. Notably, the biological basis of cannabis floral organs remains underexplored, especially when considering the substantial potential applications of the plant. Figure 1, derived from a core collection of Web of Science literature (12,444 records), illustrates the research trends in cannabis, with ‘hemp’ or ‘*Cannabis sativa*’ as the search terms. Using VOSviewer software (version 1.6.20), a keyword co-occurrence network was generated [12]. The figure clearly highlights that current research is concentrated in applied fields such as ‘composite’, ‘crop’, ‘cannabidiol (CBD)’ and ‘management’. In contrast, the ‘flower’ theme (marked by a black hexagon) related to reproductive development has small nodes and few connections. This observation points to a significant gap in the depth and comprehensiveness of flowering regulation research, which constitutes a major knowledge gap in the field.

In response to this gap, this paper systematically reviews the biological characteristics, key regulatory factors, genetic basis, and molecular mechanisms of cannabis floral organs. It also provides an overview of the industrial applications and future research directions of flowering regulation, offering a foundation for both advanced research and industrial practices in this field.

## 2. Research Progress on Biological Characteristics of Hemp Flower

### 2.1. Flower Phenotype and Sex Dimorphism

**Figure 2:**
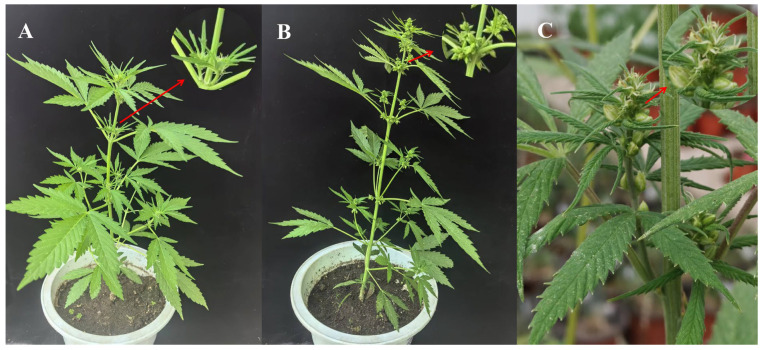
Male and female flower morphology of hemp. Note: (**A**): Dioecious male flowers, (**B**): Dioecious female flowers, (**C**): Monoecious male and female flowers. The red arrow points to a magnified inset showing a detailed view of the specified region. Photos were taken in the greenhouse of Institute of Industrial Crops, Heilongjiang Academy of Agricultural Sciences by the corresponding author Lili Tang.

Cannabis is primarily dioecious (Figure 2A,B), with a small proportion of monoecious (Figure 2C) or hermaphroditic individuals [13]. The morphology of monoecious plants resembles that of female plants before male flowers develop. Notable morphological differences exist between the male and female flowers of cannabis. These distinctions are evident not only in the appearance, color, and inflorescence arrangement but also in the more detailed features of bract morphology, calyx characteristics, and reproductive organ structure. These variations directly influence pollination efficiency and reproductive function. This pronounced sexual dimorphism emerges during the transition from vegetative to reproductive growth (i.e., floral induction) and is tightly regulated by both genetic and environmental factors [14]. Significant differences in color, inflorescence structure, bract morphology, and reproductive organs between male and female flowers are summarized in Table 1. For instance, male flowers typically have a lighter color and fluffy inflorescences adapted for wind pollination, while female flowers are characterized by dark green bracts and a compact structure designed to protect ovules. These differences have a direct impact on pollination efficiency and reproductive function. Table 1 outlines the specific differences in key traits and their potential biological significance, offering a foundation for the subsequent analysis of flowering characteristics and gender differentiation. The following descriptions are based on classical cannabis botany [15] and related research [13].

### 2.2. Flowering Characteristics and Its Effects

The diameter of cannabis pollen grains is approximately 30 microns, primarily dispersing through wind. A single flower can release over 350,000 pollen grains into the environment [16]. Under natural conditions, male flowers typically enter the flowering period 4–6 weeks after plant growth, while female flowers begin to bud 1–2 weeks, or even 3–4 weeks, after male flowers open. The flowering period of different genotypes can vary significantly, spanning 34–50 days [17]. Although this flowering timing is ideal for female flower pollination—since male flowers open first, maintaining high pollen density—it may also lead to pollen loss or inactivation due to extreme weather or a prolonged flowering interval that does not align with the reproductive cycle, thus reducing pollination and seed-setting success. This discrepancy may stem from flowering regulatory genes located on sex-related chromosomes (e.g., proteins on the male Y chromosome that promote flowering gene expression and proteins on the female X chromosome that inhibit it) or be indirectly regulated by plant hormones controlling sex differentiation [18].

The flowering process in cannabis follows a sequential and continuous progression: flower induction begins with the formation of undifferentiated primordia in the axil of the stipule, a process with neutral daily characteristics that can occur independently of photoperiod. This is followed by axillary bud formation and branch transformation, laying the foundation for inflorescence development. The next stage marks the initial phase of inflorescence flowering, characterized by dense flower clusters at stem tips and axillary buds, triggered by short-day induction. The final stage involves the apical meristem ceasing infinite growth and forming terminal flowers, although not all varieties reach this stage. This developmental framework outlines the complete path from local flower bud induction to the formation of the entire inflorescence structure, which is critical for accurately defining the flowering stage and assessing the reproductive development integrity of the variety [18].

Additionally, the flowering period directly impacts the accumulation of secondary metabolites. Pollination results in a significant reduction in the total cannabinoid concentration within the inflorescence, while preventing pollination stimulates the formation of new flowers and increases cannabinoid production [19]. Therefore, regulating the natural flowering differences between male and female flowers is key to improving breeding success and stabilizing the accumulation of medicinal compounds.

### 2.3. Flower Differentiation and Gender Plasticity

Sex differentiation in hemp is a key step in the flowering process, directly determining the type and function of floral organs. This differentiation primarily occurs during the transition from vegetative to reproductive growth, especially triggered by photoperiod changes (from long-day to short-day conditions). Within 1 to 2 weeks after receiving the photoperiod signal, the plant’s floral primordia begin to develop in a gender-specific direction: male plants form small globular clusters at the leaf axils, while female plants differentiate into pistil primordia with stigma [20]. Although sex determination follows a genetic basis (the XY system) [21], factors such as photoperiod, environmental stress (e.g., nutrient, water, damage), and endogenous hormone levels (e.g., ethylene, gibberellin) all significantly influence final sex expression during this critical period [22]. Notably, the sex phenotype in cannabis remains plastic after differentiation, allowing for sex conversion through exogenous interventions. For instance, ethylene analogues and 6-benzyladenine(6-BA) can induce feminization, while gibberellin (GA), silver ions (such as AgNO_3_), and silver thiosulfate can induce masculinization. This ability to manipulate sex expression is a key technology for regulating flowering and breeding practices, opening the door for artificial regulation of flowering and sex ratios in cannabis populations.

## 3. Effects of Photoperiod on Flowering Period of Cannabis

Light is a key environmental factor influencing flowering. Most cannabis varieties are short-day dependent, and their flowering process involves a complex interaction between photoperiod (time control) and light quality (signal regulation) [7,23]. Hemp photoperiod types are classified into three categories: short-day dependent, long-day dependent, and photoperiod-insensitive. The flowering induction of hemp varies with the duration of sunlight exposure, leading to distinct characteristics in variety distribution and cultivation application (Table 2). Among these, short-day dependent types are most common and suited for temperate seasons, while photoperiod-insensitive types are preferred in controlled environmental agriculture due to their ease of management.

### 3.1. Physiological Basis of Synergistic Regulation of Photoperiod and Light Quality

The regulation of photoperiod on cannabis flowering involves intricate physiological mechanisms, where light quality modulates the flowering period via the phytochrome signaling pathway. This pathway integrates circadian rhythms and light signals (via phytochromes and cryptochromes) to trigger flowering under long-day conditions [25], with photoperiod duration exerting a synergistic effect on the flowering process. Specifically, blue light delays flowering while enhancing light reaction efficiency [26]. The combination of red light (600–700 nm) and far-red light (700–750 nm) induces flowering and promotes leaf and stem elongation, while red light alone suppresses flowering [27]. Far-red light influences flowering timing primarily by altering the red to far-red light quantum flux density ratio (R:FR) [28]. A decrease in the R:FR ratio signals plants to interpret their environment as shaded, initiating a shade avoidance response. A key feature of this response is the acceleration of flowering to complete reproduction before competition intensifies [29]. This mechanism also explains the impact of high planting density on flowering. At high density, upper leaves absorb red light and transmit far-red light, reducing the R:FR ratio at the canopy’s lower layers, which in turn triggers early flowering [30]. These findings align with a two-year field study in central Italy, where seven hemp genotypes were grown at three planting densities (40, 80, and 120 plants m^−2^). The study reported that the vegetative phase (emergence to flowering) was significantly shorter at high density (120 plants m^−2^) compared to low density across all genotypes [9].

While previous studies have primarily focused on fiber yield, seed yield, or cannabinoid accumulation, there has been limited research on the specific regulation of flowering. However, evidence suggests that light quality plays a pivotal role in fine-tuning the transition from vegetative to reproductive growth under a consistent photoperiod. For instance, Cui et al. provided indirect evidence for light quality regulation of flowering. In their experiment, plants advanced to flowering on the 35th day, with vegetative growth significantly inhibited by a treatment group exposed to 12 h of light with a specific light quality (e.g., LEDA-FR:R:B:G = 8:9:3:12) [31]. Notably, the weak light during the dusk period has also been shown to impact cannabis flowering initiation. Zhang et al. demonstrated in a field experiment in Florida that the “effective photoperiod,” which incorporates dusk light, more accurately predicted cannabis flowering time than the “apparent photoperiod” from sunrise to sunset. This highlights the importance of considering the dusk period for high-precision flowering regulation [6].

### 3.2. Effects of Photoperiod on Flowering Initiation, Process, and Duration

Photoperiod effects on flowering exhibit significant varietal differences and dose-dependent responses. Zhang et al. systematically examined the flowering responses of 15 seed cannabis and 12 fiber cannabis varieties under 11 distinct photoperiods (12 to 18 h). The study was conducted in seven identical controlled rooms, with 5–10 replicates per cultivar. Data were analyzed using a restricted maximum likelihood mixed model, and means were separated by Tukey’s HSD test at *p* ≤ 0.05. They found that all seed varieties could flower under a 12-h photoperiod, while none bloomed under 18 h. Among them, Cherry Wine-CC, PUMA-3, and PUMA-4 exhibited the shortest critical photoperiod, ranging from 13 h and 45 min to 14 h. When the photoperiod exceeded 13 or 14 h, flowering in seed varieties was generally delayed by 1–2 or 7–8 days, respectively. For fiber varieties, flowering was delayed by 1–3 days when the photoperiod exceeded 14 h. The study also revealed that a mere 15-min photoperiod difference could significantly affect flowering initiation in some seed varieties, indicating that certain cannabis varieties are highly sensitive to photoperiod changes. Furthermore, the sensitivity of male plants to photoperiod was generally higher than that of female plants [6].

Different photoperiod treatments directly influence the appearance time of flowering initiation markers. Peterswald et al. tested three varieties—Northern Lights, Hindu Kush, and Cannatonic—and found that all three began the flowering process on the 46th day after cloning, marked by the appearance of pistils. Extending the photoperiod (14L:10D) induced some plants to produce leaf hairs, a marker closely linked to reproductive development, as early as 34 days after cloning. In contrast, shortening the photoperiod (10L:14D) delayed the appearance of both pistils and leaf hairs, suggesting that longer dark periods do not necessarily promote earlier flowering [32].

Varietal responses to photoperiod vary. A simulation study of major hemp varieties in China quantified these differences. Southern varieties (e.g., Yunma 1 and Yunma 7) were more sensitive to photoperiod. Northern varieties (e.g., Jinma No.1 and Qingdama No.1), characterized by a shorter basic nutritional period, were less sensitive to photoperiod and flowered earlier under suitable conditions. Cannatonic, a high-CBD cultivar, exhibited the latest pistil formation in all treatments but had visible leaf hairs in 100% of plants the earliest. Photoperiods longer than 13.2 h extended flowering duration, while 12 h of light resulted in the fastest flowering. Under 14 h of light, Northern Lights and Hindu Kush varieties may not complete the reproductive stage and mature slowly, in contrast to the more rapid maturity of Cannatonic varieties [32].

Further experiments with other varieties confirmed the regulation of photoperiod on flowering. The flowering initiation of ‘Incredible Milk’ (IM) was delayed by approximately 1.5 days under 13-h light treatment, and the plants were harvested on the 58th day after the start of the photoperiod treatment (harvest criteria: >95% stigma browning and 10% ambering of leaf hairs). The Gorilla Glue (GG) cultivar did not experience delayed flowering initiation under either 12-h or 13-h light treatments and was harvested on the 72nd day (harvest criteria: >95% stigma browning and fan leaf senescence) [33].

The flowering duration of hemp varies depending on the variety and growth conditions, typically ranging from 7 to 12 weeks [34]. Under 12 h of light treatment, stigma browning and leaf hair ambering occurred earlier and progressed faster, while early inflorescence development was slightly delayed under 13-h light treatment [33]. Yep, B et al. used ‘days in the flowering stage (DFS)’ as the time unit and found that the Nordle and Sensi Star varieties reached maturity at approximately 82 and 87 days, respectively, under 12-h light treatment [35].

### 3.3. Objectives and Matching Measures of Photoperiod Regulation

Targeted photoperiod management strategies can be adopted based on different production objectives. For photoperiod-sensitive varieties, short-day treatments of approximately 12 h can effectively advance flowering and accelerate stigma browning and leaf hair ambering [33]. In contrast, photoperiods longer than 13.2 h (e.g., 14 h of light and 10 h of darkness, or more extreme conditions like 22 h of light and 2 h of darkness, which may include 10–11 h of natural light) can delay pistil emergence and leaf hair formation, prolong the flowering period, and, in some cases, prevent flowering entirely [36]. Additionally, inducing flowering relies on maximizing natural short-day conditions. For example, in Florida’s Apopoca area, where sunlight lasts about 10 h and 20 min, flowering can be effectively induced after sufficient vegetative growth (e.g., 58 days) [37].

Different strategies are needed for distinct photoperiod types (detailed in Table 2): for photoperiod-insensitive varieties, flowering can be regulated through temperature management (e.g., early sowing and low-temperature conditions); for photoperiod-sensitive varieties, adequate vegetative growth under long-day conditions is required to prevent premature reproductive growth [38]. Timely sowing or transplanting is a fundamental and key measure to align with local natural photoperiods and achieve precise flowering regulation. Studies in various ecological regions, such as southern Italy [39], Kunming, China [40], and Florida, USA [41], have demonstrated that selecting the optimal sowing date can prevent premature flowering induced by short-day conditions or relatively early flowering resulting from late sowing/planting, thus optimizing reproductive growth.

Moreover, the application of photoperiod regulation is particularly valuable in crossbreeding, offering a key technology to address the issue of ‘flowering period mismatch.’ For instance, Somody and Molnár successfully induced synchronous flowering in their parent plants by setting up a shading system and applying a 12-h short-day treatment to parents with a flowering period of approximately 60 days, enabling controlled artificial pollination. This suggests that actively ‘scheduling’ flowering in greenhouses or controlled facilities using photoperiod manipulation is an effective strategy to overcome reproductive isolation between parents with different genetic backgrounds, thereby accelerating the breeding process [42].

## 4. Effects of Plant Nutrition and Plant Hormones on the Flowering Period of Hemp

### 4.1. Regulating Effect of Mineral Elements

The supply levels and proportions of macronutrients are central to regulating the flowering period of cannabis [43]. The regulation of nitrogen, phosphorus, and potassium is particularly significant:

Nitrogen (N): Nitrogen levels profoundly influence the transition between growth stages. Adequate nitrogen promotes growth, while excessive nitrogen can disrupt hormone balance, leading to abnormal flowering. In a Mediterranean field experiment evaluating seven genotypes under two nitrogen levels (50 and 100 kg N·ha^−1^), under low nitrogen levels (50 kg N·ha^−1^), the vegetative period lasted an average of 69 days, slightly longer than the 67 days observed under high nitrogen levels (100 kg N·ha^−1^), though this effect varied across genotypes [9]. Adjusting the nitrogen-to-potassium ratio is a common strategy for regulating the growth stage. For example, higher nitrogen fertilizer is required to increase fiber yield and prolong vegetative growth [44].

Phosphorus (P): Phosphorus plays a pivotal role in inducing flowering. De Prato et al. [45] demonstrated that adding phosphate rock powder could advance the flowering period of both female and male plants of the tropical variety ECO-MC16 by 10 and 15 days, respectively.

Potassium (K): Potassium demand peaks during the critical reproductive growth phase (from squaring to flowering) to support inflorescence development and metabolic activities [46]. About 35 days after flowering, the demand for phosphorus and potassium increases significantly to support processes such as inflorescence expansion and resin synthesis [35].

In addition to macronutrients, trace elements such as boron, zinc, and copper are essential for reproductive development. Boron deficiency can lead to abnormal reproductive organs [47], zinc deficiency can inhibit flowering [48], and copper deficiency can reduce pollen viability [49]. A key future research direction is the systematic exploration of the specific mechanisms through which trace elements regulate cannabis flowering.

### 4.2. Application of Plant Growth Regulators and Exogenous Hormones

Plant growth regulators and hormones play a critical role in regulating the flowering time in cannabis, directly influencing flowering onset and indirectly affecting the process by modulating the sex ratio.

Flowering time is defined as the moment when the first single flower appears in the axil of the petiole, a standard established by Zhang Xiaoyu [8]. In the phytohormone experiment of that study, plants from the BPC2-ED2 mutant line, MIKC1-ED7 mutant line, and non-transgenic DMG12 were selected, with 18 plants per treatment group (6 plants per genotype). The application of plant hormones was timed to occur when the first leaf bud appeared, which was 8–10 days earlier than the anticipated flowering time of cannabis, to explore their regulatory effects on flowering. Hormone solutions (Ethrel, abscisic acid (ABA), 6-BA, silver thiosulfate (STS)) or deionized water (control) were sprayed every 8 days. Three flowers per plant were randomly sampled, and each dataset contained 18 biological replicates. Statistical significance is indicated in the original figure legends. Treatment with the ethylene analogue Ethrel (0.69 mmol·L^−1^) resulted in an average flowering date that was 4.23 days earlier, while spraying the ethylene inhibitor STS delayed flowering initiation by an average of 3.22 days [8]. GA is another key hormone in the regulation of flowering. Short-day conditions reduce the levels of GA4 and auxin in shoot tips, promoting the development of dense inflorescences, whereas long-day conditions or exogenous GA application increase GA levels and inhibit inflorescence development. This suggests that GA plays a critical role in sexual reproduction, inflorescence development, and the formation of inflorescence structures in female cannabis by mediating photoperiod signals in inflorescence tissues [50]. In fiber hemp production, exogenous GA application to inhibit flowering and prolong vegetative growth is an effective strategy to increase fiber yield.

Exogenous treatments can also effectively alter the sex expression of cannabis, thereby influencing the group’s flowering period.

Induced feminization: Prior to sex differentiation, low concentrations (0.1–1.0%) of wood vinegar root application can increase the likelihood of monoecious individuals, while higher concentrations (1.0–1.5%) significantly increase the number of female plants [51]. Yu M.D. et al. [52] sprayed 0.27 mmol·L^−1^ and 0.53 mmol·L^−1^ 6-BA on the leaves of Longdama No.5 at the four-leaf stage. The results showed that 6-BA treatment promoted feminization in cannabis, with higher concentrations of 6-BA leading to a higher female rate.

Induced masculinization: Under short-day conditions, 3 mmol of silver thiosulfate can efficiently and stably induce female plants to produce male flowers after three foliar sprays (at 7-day intervals), providing the optimal treatment for producing all-female seeds [53]. GA can also induce female plants to produce male flowers, while ABA can partially or completely inhibit this phenomenon depending on the concentration [22]. Spraying 0.4 mmol·L^−1^ AgNO_3_ 80 days after sowing, and 0.58 mmol·L^−1^ GA3 60 days after sowing, significantly reduced the female-to-male ratio [54]. Additionally, exogenous zearalenone treatment increased the male-to-female ratio in cannabis [55].

Notably, the underlying molecular mechanisms through which these exogenous hormones and chemical reagents regulate flowering time and sex differentiation in cannabis remain to be elucidated. However, drawing upon research from other cultivated plants, it is plausible to speculate that these signals may eventually converge into and modulate the core molecular network governing cannabis flowering. For instance, in radish (Raphanus sativus), exogenous gibberellin (GA) treatment promotes bolting and flowering under insufficient vernalization by significantly upregulating the expression of flowering integrators *RsFT* and *RsSOC1* [56]. Conversely, ABA, a stress-responsive hormone, antagonizes GA signaling and inhibits *FT* expression, leading to delayed flowering. In Arabidopsis, ABA directly activates the transcription of the flowering repressor *FLC* through its signaling component, the transcription factor ABI4, which subsequently represses *FT* expression and thus delays flowering [57]. Ethylene signaling extensively interacts with photoperiod and temperature pathways, affecting the stability of genes such as *CO* and *FT*. Classical studies have demonstrated that ethylene can reduce active GA levels and promote DELLA protein accumulation via the *CTR1*/*EIN3* pathway, thereby suppressing the expression of flowering integrators *LFY* and *SOC1* and delaying flowering [58]. Based on these findings, we propose that in cannabis, the male-promoting effect of GA, the inhibitory effect of ABA on GA-induced responses, and the sex-reversal function of ethylene signaling inhibitors (e.g., AgNO_3_) are likely mediated through the regulation of key flowering genes such as *CsFT*/*CsHd3a* and *CsPRR37* (detailed in Section 6 and Section 7). Systematically elucidating the interaction networks between hormone signals and these core flowering pathways will be crucial for bridging agronomic regulation strategies with molecular breeding approaches and represents an important direction for future research.

## 5. Effect of Stress on the Flowering of Hemp

Currently, there is limited research directly correlating stress with the regulation of flowering in cannabis, with most related studies focusing on its impact on fiber yield or secondary metabolite production. However, biotic and abiotic stresses, as key factors influencing growth and development, are likely to indirectly regulate flowering by interfering with physiological and metabolic processes [59]. For instance, it has been suggested that factors such as heavy metal pollution, temperature fluctuations, and pathogen infections could potentially alter flowering time, progression, or even reproductive success in cannabis, although direct mechanistic evidence remains scarce.

### 5.1. Abiotic Stress

Regarding abiotic stress, existing studies primarily report observational correlations rather than elucidating definitive regulatory mechanisms. For example, exposure to high concentrations of cadmium (25 mg·L^−1^) was reported to induce premature senescence in the medicinal cannabis variety Purple Tiger, causing plant death before flowering could be completed [24]. This observation indicates that severe cadmium toxicity can prevent the flowering process, but it does not clarify whether sub-toxic levels can directly modulate flowering timing. Temperature stress, particularly low temperatures, has been associated with an extended duration required to complete the basic vegetative phase, which could indirectly delay the onset of flowering [7,60]. Additionally, an unconventional negative temperature differential (higher nighttime than daytime temperatures) was shown to inhibit flower development in a low-Δ^9^-tetrahydrocannabinol (Δ^9^-THC) cannabis strain, manifesting as delayed flowering, reduced flower number, and inferior quality [61]. Collectively, these studies point to potential links between abiotic stress and flowering phenotypes, but further research is needed to uncover the direct molecular or hormonal pathways involved.

### 5.2. Biotic Stress

Research directly connecting biotic stress to flowering regulation in cannabis is even more limited. An interesting correlation has been noted in the context of powdery mildew, where disease incidence appears to vary with the plant’s maturity stage (i.e., the flowering process) [5]. This correlation suggests a possibility that flowering time itself may influence susceptibility to pathogens, hinting at a potential bidirectional relationship between biotic stress and reproductive development. However, explicit evidence on whether pathogens can actively modulate the host’s flowering pathways is currently lacking and warrants future investigation.

In summary, although direct evidence is limited, preliminary observations have linked various biotic and abiotic stresses to the flowering period in cannabis. The underlying mechanisms likely involve stress signals disrupting endogenous hormone homeostasis (e.g., inducing ABA accumulation or inhibiting GA biosynthesis), thereby interfering with the normal function of core flowering regulatory networks. For example, in Arabidopsis and rice, stresses such as drought and cold significantly suppress the expression of *FT* orthologs (e.g., *FT* in Arabidopsis, *Hd3a*/*RFT1* in rice) through ABA-dependent or -independent pathways, involving regulatory modules such as NF-Y transcription factors and miRNAs (e.g., miR169, miR172), ultimately delaying flowering [62]. Pathogen infection (e.g., by Pseudomonas syringae) can trigger salicylic acid (SA) and ABA signaling pathways, enhancing stomatal defense and suppressing the expression of flowering integrators like *FT* and *SOC1*, while simultaneously upregulating the flowering repressor *FLC* to delay flowering. This reflects a survival strategy wherein plants prioritize resource allocation to defense responses under biotic stress [63]. Currently, in cannabis, it remains unclear whether and how stress signals regulate pathways such as *CsFT*, *CsPRR37*, or the *miR156-SPL* module via hormonal intermediaries (e.g., ABA/GA balance). Future research should integrate multi-omics analyses (e.g., transcriptomics, hormonomics) under stress conditions with phenotypic validation using key gene mutants or overexpression lines, to map the molecular roadmap from “Stress perception leads to hormone remodeling, which in turn results in flowering time adjustment.”. This effort is not only crucial for deciphering the environmental adaptability of cannabis but will also provide theoretical targets for stabilizing flowering and yield through cultivation management in the context of climate change.

## 6. Genetic Basis of the Flowering Time of Hemp

### 6.1. Genotypic Differences in Flowering Time and Germplasm Resources

Significant differences in flowering time exist among different cannabis genotypes, providing a foundation for genetic improvement and cultivation regulation. Numerous studies have confirmed this broad diversity:

Stack et al. [5] conducted replicated field trials with 30 high-cannabinoid hemp cultivars across two sites in New York State and observed substantial variation in flowering time. A significant effect of cultivar on flowering date was reported (*p* < 0.001), with early- and late-flowering individuals segregating within some seeded populations. Based on flowering time, they categorized the varieties into five groups: daily neutral varieties (e.g., Kayagene varieties), extremely early/early flowering varieties (e.g., early flowering individuals of Umpqua, Rogue, and Deschutes), medium flowering varieties, and late flowering varieties (e.g., Late Sue flowering in early October). Variations in flowering time were also observed within certain varieties. For instance, in Umpqua and Deschutes, the ratio of early flowering to late flowering individuals is approximately 1:1, while in Rogue, it is approximately 1:3.

In a replicated two-year field trial in central Italy that tested seven monoecious hemp genotypes under different plant densities and nitrogen levels, early flowering genotypes (e.g., Fedora17, Felina32, Ferimon, Uso31) exhibited a shorter time from emergence to flowering, approximately 60 days. In contrast, middle and late flowering genotypes (e.g., Epsilon68, Futura75, Santhica27) had longer vegetative growth periods and later flowering times, which corresponded with their higher stem yields [9].

**Figure 3:**
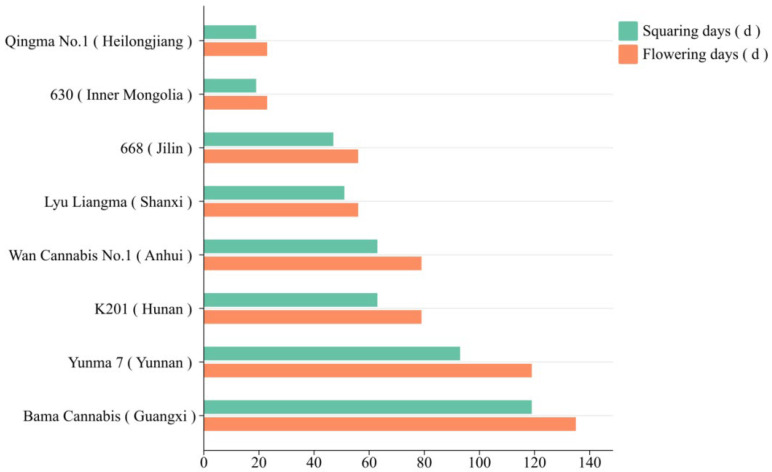
Variation in days to flowering among representative Chinese hemp germplasm accessions under short-day conditions. Note: The data are compiled from Li [64]. Among them, 668 and Lyu Liangma are photoperiod-insensitive, while the others are photoperiod-sensitive.

A systematic investigation of Chinese germplasm resources also revealed substantial flowering time diversity. Li [64] conducted a 148-day field survey of 179 accessions planted in Changsha, Hunan under short-day (SD) conditions. The survey, focusing primarily on male plants due to their earlier and more uniform flowering, recorded days to budding and flowering. Results showed a broad range: the earliest flowering occurred at 23 days after sowing (DAS), while the latest was at 135 DAS. Figure 3 lists the flowering times of some representative varieties. These germplasms provide key experimental materials for cloning and functional studies of flowering-related genes. Chen et al. [65] classified Chinese cannabis into five groups through population genomics analysis, finding that wild populations (e.g., from Northeast China and Xinjiang) typically flower earlier than cultivated populations, highlighting the relationship between genetic differentiation and flowering adaptability. This conclusion was based on whole-genome resequencing of 52 cannabis accessions, including 21 newly sequenced Chinese accessions and 31 publicly available datasets. Population structure was determined using principal component analysis and model-based clustering, while key phenotyping of flowering time was conducted under both natural short-day conditions in Kunming and controlled long-day/short-day treatments in growth chambers.

### 6.2. Genetic Mapping of Key Flowering Gene Loci

With the advancement of molecular marker technology and genome-wide sequencing, genome-wide association studies (GWASs) and quantitative trait locus (QTL) mapping have emerged as effective methods for identifying gene loci associated with flowering time [66]. In a GWAS involving 123 hemp germplasm accessions, Petit et al. [67] performed phenotypic evaluations across three European locations (Italy, France, and the Netherlands) using approximately 600,000 SNP markers. They identified six flowering time-related QTLs (validated across environments), encompassing 33 transcripts predicted to be involved in flowering regulation, including those related to light perception and signaling (e.g., cryptochromes, phytochromes) and miRNA pathways. Statistical analyses were conducted using a mixed linear model (corrected for kinship) with Bonferroni correction for multiple testing (significance threshold: −log_10_P ≥ 4.047).

Furthermore, the natural variation in photoperiod insensitivity in cannabis has drawn considerable research interest, making it an ideal model for studying photoperiod regulation. Toth JA et al. [68] identified the major recessive locus *Autoflower1* (Chr1: 17.74–22.94 Mb), controlling photoperiod insensitivity, and the early flowering locus *Early1* (Chr1: 35.26–36.23 Mb) by constructing a segregating population (F2, *n* = 88) and applying bulk segregant analysis (BSA). The study developed high-throughput molecular markers that showed complete association with the phenotypes, supported by statistical tests (*Autoflower1* segregation fitted a 1:3 ratio, X2 *p* > 0.05; *Early1* fitted a 1:1 ratio, X2 *p* = 0.15). These key sites have been further confirmed and precisely mapped in recent integrative genomic analyses [18]. *Autoflower1* overcomes the limitation of short-day-induced flowering by regulating photoperiod insensitivity, allowing varieties to begin flowering under a wider range of light conditions. *Early1* directly promotes earlier flowering and accelerates the transition from vegetative to reproductive growth. In the cultivation of early-maturing varieties, these two genes form a synergistic mechanism: *Autoflower1* first alleviates the photoperiod constraints, enabling plants to enter the flowering preparation stage without relying on short-day light, while *Early1* further reduces the time from preparation to completion of flowering by accelerating the flowering process. This gene-level synergy provides a clear target for optimizing the growth cycle and enhancing environmental adaptability in cannabis.

Subsequently, Dowling et al. [69] constructed a hybrid population (Felina 32 × Finola) and, using bulked segregant analysis (BSA) combined with whole-genome sequencing (QTL-seq) of early- and late-flowering bulks from F2 and F3 populations, identified *Autoflower2*, a 0.5 Mbp locus on chromosome 8 significantly associated with photoperiod-insensitive flowering. The locus contains a tandem-duplicated CsFT1 gene, with structural (SNPs and InDels) and expressional differences between photoperiod-sensitive and insensitive cultivars, supporting its role in daylength-independent flowering.

*CsPRR37*, a pseudo-response regulator associated with *Autoflower1*, functions as a strong flowering inhibitor under long-day conditions. A G-to-T mutation at the donor splicing site of its second exon disrupts normal RNA splicing, producing truncated proteins with loss of function [70]. This mutation relieves the inhibition of the florigen gene *CsHd3a*, leading to premature accumulation of *CsHd3a* under long-day conditions and early flowering [71]. Transient transgenic experiments confirmed that functional *CsPRR37* can re-inhibit *CsHd3a* and delay flowering [71].

*CsHd3a*/*FT*-like genes (e.g., *CsFT3* on ChrX) are core florigen orthologs, with natural variation correlating with flowering time [64,72]. Li Zheng [64] cloned *CsHd3a* from the early-flowering variety ‘Q1’, obtaining a 543-bp CDS encoding a 180-amino-acid protein belonging to the *PEBP* family. qRT-PCR analysis (using DHS2 as reference) revealed that *CsHd3a* was predominantly expressed in leaves, with minimal expression in roots and stems. Under SD conditions (8 h light/16 h dark), *CsHd3a* expression in ‘Q1’ showed a diurnal rhythm (peaking at 8:00 AM) and was significantly higher than in the late-flowering variety ‘Y7’ (*p* < 0.01), while under LD conditions, its expression was barely detectable. Sequence comparison between ‘Q1’ and ‘Y7’ revealed amino acid substitutions within the conserved *PEBP* domain, suggesting a role in flowering time differences.

Additionally, key regulatory regions such as the *FT3*/*CEN1* region on chromosome X (~85–100 Mb) regulate flowering and inflorescence development [18]. Notably, photoperiod-insensitive traits are also present in wild cannabis resources (e.g., C. ruderalis), where flowering is primarily regulated by plant age rather than photoperiod [73], offering an important natural resource for breeding and genetic research. The functions and characteristics of the key genetic loci mentioned above are summarized in Table 3.

## 7. Molecular Mechanism of Flowering Regulation

### 7.1. Core Genes and Networks of Photoperiod Pathway

*FT* (*FLOWERING LOCUS T*) is a core gene involved in flowering regulation, with well-established functions in *Arabidopsis* and other plants. In cannabis, *FT* also plays a critical role in flowering regulation, being induced by long-day and short-day conditions in wild and cultivated cannabis, respectively, to promote flowering [65].

For traditional short-day-dependent varieties, 10–12 h of darkness can trigger the phytochrome signaling pathway, induce the expression of the florigen gene *CsHd3a* (*FT* homolog), and initiate reproductive growth [74]. Shading treatments (12 h of light: 12 h of darkness) in cultivation can achieve precise control of population flowering by synchronizing *CsHd3a* expression peaks [75].

Li Zheng [64] cloned *CsHd3a* from the early-flowering variety ‘Q1’, obtaining a 543-bp CDS encoding a 180-amino-acid protein belonging to the *PEBP* family. qRT-PCR analysis (using DHS2 as reference) revealed that *CsHd3a* was predominantly expressed in leaves, with minimal expression in roots and stems. Under SD conditions (8 h light/16 h dark), *CsHd3a* expression in ‘Q1’ showed a diurnal rhythm, peaking at 8:00 AM, and was significantly higher than in the late-flowering variety ‘Y7’ (*p* < 0.01). In contrast, under long-day (LD) conditions, its expression was barely detectable. Sequence comparison between ‘Q1’ and ‘Y7’ revealed several amino acid substitutions within the conserved *PEBP* domain, suggesting that natural variation in *CsHd3a* might contribute to flowering time differences [71]. These findings confirm *CsHd3a* as a core positive regulator (“florigen”) in the photoperiod pathway of cannabis.

In photoperiod-insensitive (self-flowering) varieties, the molecular mechanism is closely linked to changes in the *FT* pathway. *CsPRR37* acts as a key hub connecting photoperiod signals with *CsHd3a* expression: fully functional *CsPRR37* inhibits *CsHd3a* under long-day conditions, while loss-of-function mutations (e.g., G-T splice site mutation) relieve this inhibition, leading to early flowering [70]. Additionally, photoperiod-insensitive cultivars (e.g., Finola) carry tandem repeats of *CsFT1* (*Autoflower2* locus), with structural and expressional variations compared to photoperiod-sensitive varieties, contributing to daylength-independent flowering [69].

### 7.2. Other Key Regulatory Genes and Pathways

In addition to the direct upstream regulator *CsPRR37* in the *FT* core pathway, other gene families and molecular networks contribute to a more complex flowering regulation system [76].

The *CO-like* gene family exhibits circadian rhythmic oscillations, with peak expression coinciding with the critical period for photoperiod-induced flowering [77]. Genome-wide identification of the *CsCOL* family confirmed its circadian rhythm expression characteristics and functional differentiation: 10 *CsCOL* genes are preferentially expressed in leaves, *CsCOL13* in stems, and *CsCOL2* and *CsCOL3* in female flowers. Under SD and LD conditions, most *CsCOL* genes show diurnal expression fluctuations, with *CsCOL4* and *CsCOL11* highly expressed in early-flowering varieties (‘Q1’,‘H7’) and *CsCOL6*, *CsCOL7*, *CsCOL9*, and *CsCOL12* in late-flowering ones (‘Y7’,‘BM’) [64].

*CsFD*, similar to *AtFD* in Arabidopsis, promotes flowering and may contribute to earlier flowering in male plants. *CsREM16*, a female-biased gene (expressed at both vegetative and flowering stages), is a hub gene in female-specific module III (identified via WGCNA), which consists of 154 genes (75% located on the X chromosome) highly expressed in female plants, suggesting roles in reproductive development and flowering regulation [78].

Cannabis flowering time and inflorescence structure are regulated by multiple pathways: (1) Photoperiod pathway: *PEBP* family gene *FT3* is highly expressed under short-day conditions to promote flowering, while *CEN1* acts as an inhibitor; (2) Temperature-dependent pathway: *FLC*-like gene delays flowering by inhibiting *SOC1*, while high temperatures activate *FT* via *PIF4* to accelerate flowering; (3) Age pathway: miR156-SPL9 regulates the vegetative-to-reproductive growth transition, while miR172 promotes flowering by targeting AP2-like proteins [18,79].

**Figure 4:**
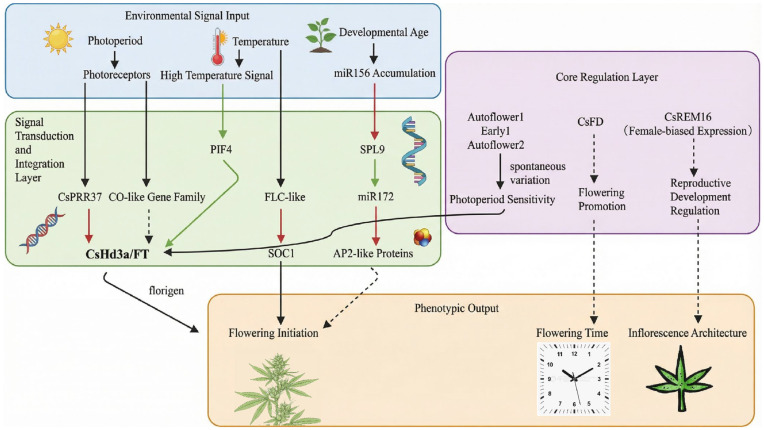
Integration diagram of the core molecular mechanism of hemp flowering regulation. Note: Red arrows indicate inhibition or repression; green arrows indicate promotion or activation; black arrows represent connections or processes. Solid line arrows indicate confirmed or well-established regulatory relationships, while dotted line arrows represent speculated or not fully confirmed relationships.

In summary, cannabis flowering regulation is a complex molecular network involving multiple signaling pathways, including photoperiod, temperature, and age. The core genes and regulatory relationships can be integrated as shown in Figure 4.

## 8. Summary and Prospect

As a core technology in cannabis cultivation management, flowering regulation directly impacts the growth rhythm, material accumulation, and industrial benefits of crops. The development of models based on environmental factors provides a scientific foundation for accurately predicting flowering time, determining optimal sowing dates, and designing introduction schemes [39]. This approach facilitates the optimization and enhancement of industrial benefits across multiple dimensions, such as fiber yield and quality, the extraction efficiency of medicinal components, the stability of seed supply, and the flexibility of planting arrangements.

In fiber production, delaying flowering through flowering regulation technology can effectively inhibit stem lignification [60], allowing plants to fully accumulate lignin and cellulose during the vegetative growth stage. This leads to significant improvements in fiber yield and quality, meeting the demands of high-end textile manufacturing. Regarding the extraction of medicinal components, flowering is a critical period for the synthesis and accumulation of medicinal compounds. Precise regulation is beneficial for maximizing the synthesis of target medicinal components, such as CBD, at specific stages [80,81], while minimizing the proportion of undesirable compounds. Studies have shown that cannabinoid levels, particularly CBD, generally increase as flowering progresses in various cannabis varieties [82]. Additionally, the biological activity of flower extracts is closely linked to metabolite accumulation and gene expression [83]. For seed production, proper regulation of the flowering period ensures that seeds fully develop and mature in a suitable environment, thereby enhancing seed plumpness, germination rates, and nutritional value [44]. Research on hemp inflorescences and proteins as new food sources is also advancing [84,85]. Furthermore, flowering regulation can improve the flexibility of cropping systems, enabling multiple cropping, rotation, or intercropping with other crops, thus enhancing land use efficiency [86].

It is crucial to acknowledge that research and applications involving *Cannabis sativa* L. are subject to complex and varying legal frameworks worldwide, particularly concerning varieties with high psychoactive cannabinoid (e.g., THC) content. A key legal distinction often hinges on the presence and concentration of (Δ^9^-THC); for instance, synthetic cannabinoid preparations devoid of psychoactive Δ^9^-THC may face different regulatory pathways compared to botanical extracts containing it [87]. All research directions proposed herein must be pursued in strict compliance with relevant national and international regulations. For industrial hemp (low THC), the focus is on agronomic and safety standards, while work on medicinal varieties necessitates additional ethical oversight regarding controlled substance handling and intended use. Therefore, future research on cannabis flowering periods should focus not only on consolidating the benefits of environmental regulation but also on translating knowledge into practical applications within appropriate legal boundaries. To guide this effort, a clear experimental roadmap is proposed:

1. Functional Genomics and Precision Breeding: Utilize CRISPR-Cas9 or other gene-editing tools to create isogenic lines with mutations in key flowering genes (e.g., *CsPRR37*, *Autoflower1*). Phenotype these lines under diverse photoperiods and environments to directly validate gene function and assess their utility in breeding programs for desired flowering traits.
2. Integrated Omics under Combined Stress: Establish controlled experiments to dissect how combined abiotic stresses (e.g., low temperature + nutrient imbalance) affect flowering. Integrate time course transcriptomic, metabolomic, and hormone profiling data from contrasting genotypes to map the regulatory networks that underlie stress-induced alterations in flowering time and development.
3. Development of Predictive Cultivation Models: Implement multi-location field trials across key production zones. Collect high-resolution data on flowering phenology, microclimates, soil conditions, and management practices. Employ this dataset to build and validate machine learning or physiological models that can accurately predict flowering time, supporting optimized sowing decisions and regional adaptation.
4. Exploitation of Germplasm and Evolutionary Insights: Systematically screen wild and cultivated germplasm collections for novel alleles of flowering time genes. Combine genome-wide association studies (GWASs) with an ecological analysis to understand how flowering time adaptation has shaped the population structure and evolution of *Cannabis sativa*.

The execution of this roadmap will require concerted interdisciplinary efforts but promises to deliver the precise control over flowering needed to optimize hemp production for fiber, medicine, food, and other emerging industries.

## Figures and Tables

**Table 1:** Characters and biological significance of male and female flowers of hemp.

Character	Male Flower	Female Flower
Color	Pale yellow-green (Light color aids pollen visibility for wind pollination)	Deep green (High chlorophyll content supports photosynthesis for seed development)
Inflorescence number & appearance	Numerous; dozens to hundreds per plant; fluffy overall (Maximizes pollen output for wind dispersal)	Fewer in number (Resource concentration for seed maturation)
Inflorescence morphology	Multiple racemes; branched; flowers on slender pedicels (Facilitates pollen dispersal)	Spicate; compact; sessile (no pedicel) flowers (Protects ovules; efficient pollen capture)
Bracts	Narrow, lanceolate; thin texture; slight protective role	Broad, ovate; rough texture; expands post-pollination to form protective seed coat
Calyx	Well-developed; 5-lobed; 2–4 cm; villous (Sepal function)	Reduced, membranous; adnate to ovary wall (Often damaged at maturity)
Reproductive organs	5 stamens; slender filaments; dangling anthers	Ovary 1-loculed, styled, with a pair of slender feathery stigmas at the top of the style, with 1 drooping ovule
Maturity indicators	Anther color: from pale yellow to brown; Filaments elongate to lift anthers	Stigma color: from light green to yellowish/reddish brown Stigma surface becomes wet (Optimal pollination window)

Note: Biological functions or significance are indicated in parentheses following the morphological descriptions.

**Table 2:** Photoperiod types of hemp.

Type	Flowering Trigger	Characteristics	Representative Varieties	References
Short-day dependent	Daylength ≤ critical value (e.g., ≤12 h)	Most common type; wild species; adapted to temperate seasonal cycles	Von, T1	[24]
Long-day dependent	Daylength ≥ critical value (e.g., ≥14 h)	Rare; high-latitude distribution; adapted to long-day environments	DMG12, YMG26, Apricot Auto, Auto CBD Alpha Explorer	[24]
Daylength-insensitive (Auto-flowering)	Independent of daylength; triggered by age/maturity	Convenient for cultivation; suitable for controlled environments or diverse latitudes	Helena	[6]

**Table 3:** Key genetic loci and genes regulating flowering time in *Cannabis sativa* L.

Gene/Locus	Chromosomal Location (cs10)	Key Phenotypic Effect	Core Functional Category	References
*Autoflower1*	Chr1: 17.74–22.94 Mb	Photoperiod insensitivity (autoflowering)	Major recessive locus	[68]
*Early1*	Chr1: 35.26–36.23 Mb	Promotes early flowering	Early flowering promoter	[68]
*Autoflower2* (*CsFT1*)	Chr8: ~0.5 Mbp region	Photoperiod-insensitive flowering	Florigen gene (*PEBP* family)	[69]
*CsPRR37*	Associated with *Autoflower1*	Loss-of-function leads to early flowering	Pseudo-response regulator	[71]
*CsHd3a*/*FT*-like (e.g., *CsFT3*)	Not specified (*CsFT3* on ChrX)	Natural variation correlates with flowering time	Florigen (*FT* ortholog)	[64,72]
*FT3*/*CEN1* Region	ChrX: ~85–100 Mb	Regulates flowering and inflorescence development	Key gene cluster	[18]
GWAS Multiloci	Multiple	Associated with flowering time	Light perception, miRNA pathways, etc.	[67]

## Data Availability

The original contributions presented in this study are included in the article. Further inquiries can be directed to the corresponding author.
